# Explainable diabetes prediction using a stacked ensemble framework

**DOI:** 10.1371/journal.pone.0352313

**Published:** 2026-09-15

**Authors:** Saher Fatima Awan, Kainat Irfan, Umair Muneer Butt, Sukumar Letchmunan, Fadratul Hafinaz Hassan

**Affiliations:** 1 Department of Computer Science, University of Management and Technology, Lahore (Sialkot), Pakistan; 2 School of Computer Sciences, Universiti Sains Malaysia, USM Penang, Malaysia; Tabriz Islamic Art University, IRAN, ISLAMIC REPUBLIC OF

## Abstract

Diabetes is a chronic disease that significantly increases the risk of serious complications such as cardiovascular disorders and kidney failure. Early detection through predictive modeling can lead to timely interventions and significantly improve patient health outcomes. Several machine learning approaches have been proposed for predicting diabetes, but the main focus has been on improving prediction accuracy, while interpretability has received limited attention. To address this gap, we present a robust and explainable machine learning framework based on a stacked ensemble model that uses Random Forest, Support Vector Machine, and Gradient Boosting as base learners and Catboost as the meta-learner. The model was trained on the PIMA Indians Diabetes dataset using a preprocessing pipeline that included standard scaling, analysis of variance (ANOVA)- F-score-based feature selection, and class balancing with the synthetic minority oversampling technique (SMOTE). The proposed ensemble model outperformed the latest methods with an accuracy of 86%. We integrated explainable AI techniques such as Local Interpretable Model-Agnostic Explanations (LIME) and Shapley Additive Explanation (SHAP) to enhance transparency, which provide both local and global interpretability by identifying the most influential features contributing to each prediction, thus supporting more informed and trustworthy decision-making in healthcare applications.

## Introduction

Diabetes is a chronic disease that is becoming severe in both industrialized and developing countries [1]. It directly affects the pancreas, rendering the body incapable of producing insulin [2]. The major factor regulating blood glucose levels in the body is insulin. Insulin deficiency causes type 1 diabetes (T1D). When the body is unable to produce sufficient insulin, it leads to type 2 diabetes (T2D) [3]. Diabetes can be caused by factors such as excessive body weight, physical inactivity, high blood pressure, and abnormal cholesterol levels [4]. Although there are many complications of it, the most frequent one is an increase in urination [5]. It is concerning because it can damage the skin, nerves, and eyes, and if not treated early, diabetes can cause kidney failure and diabetic retinopathy ocular disease.

**Fig 1:**
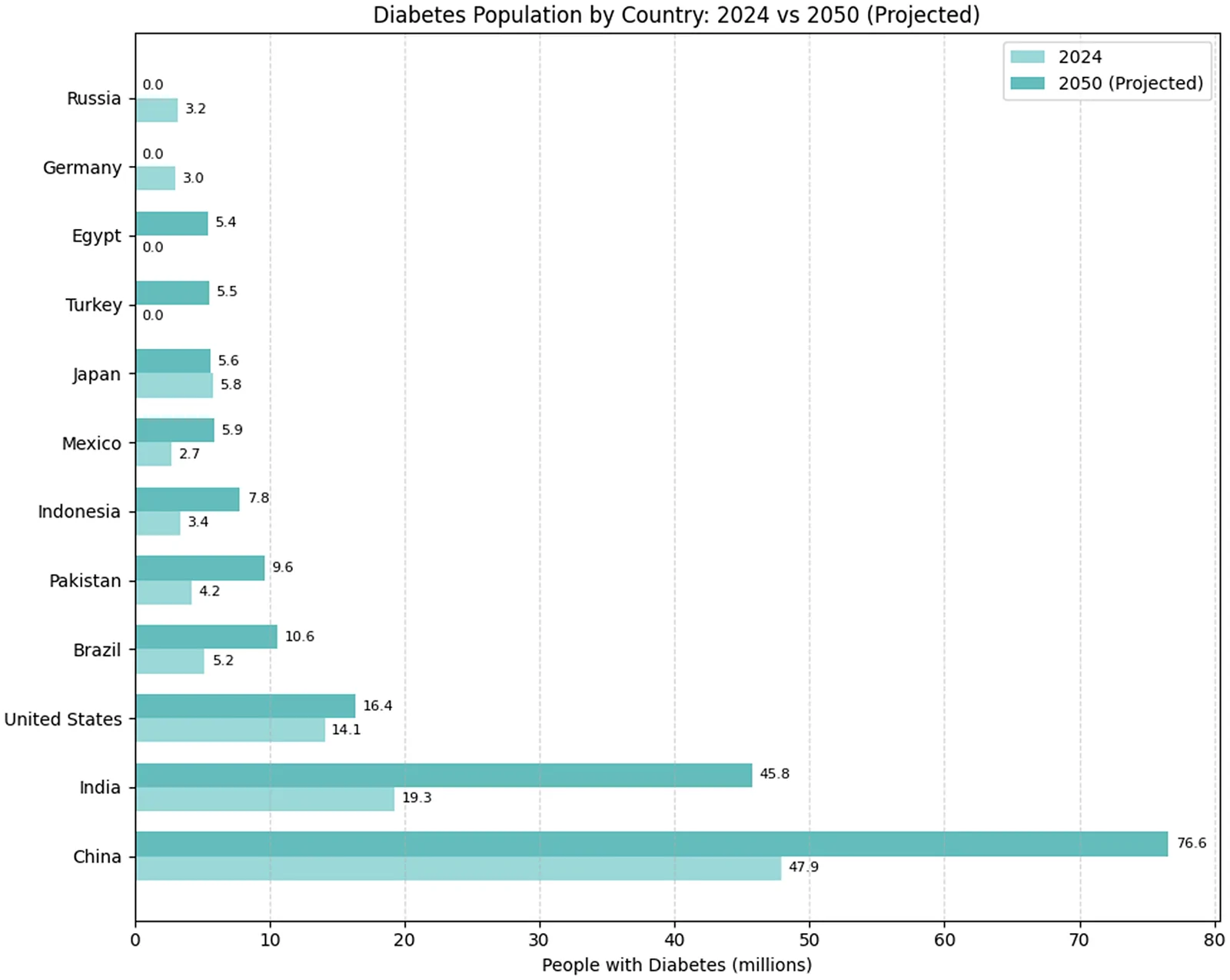
Comparison of diabetes population in 2024 and projected in 2050 across the top 10 countries.

According to IDF, 589 million people are suffering from diabetes worldwide [6]. A recent study highlighted the seriousness of diabetes by revealing that the condition affects half a billion individuals worldwide, and this figure is expected to increase by 25% and 51% by the years 2030 and 2045, as can be seen in the Fig 1, respectively [7]. is estimated that around 1.5 million individuals died directly from diabetes in the year 2012, while 2.2 million perished from cardiovascular diseases, chronic kidney disease, and tuberculosis [8]. Healthcare datasets significantly increased in complexity and size [9]. Deep learning has become a powerful alternative to machine learning models for diabetes prediction. DL models such as Recurrent Neural Networks (RNNs), LSTMs, GRUs, and hybrid architectures are increasingly employed due to their ability to automatically extract high-level features from raw data [10].

While it is evolving, the main limitation of deep learning in medical settings is its “black-box” nature. In critical domains such as healthcare, where decision-making affects lives, understanding why a model predicts a particular outcome is as important as the prediction itself. This has led to a growing emphasis on explainable AI (XAI) methods [11]. To make models interpretable, XAI methods such as SHAP (Shapley Additive exPlanations), LIME (Local Interpretable Model-agnostic Explanations), and attention mechanisms are used to interpret DL outputs [12]. The current trend in research is not just to enhance model performance, but also to integrate explainability frameworks that bridge the gap between high accuracy and clinical acceptance. The contributions of this study are:

- We propose a robust stacked ensemble model for diabetes prediction by combining Random Forest, Gradient Boosting, and Support Vector Classifier as base learners, with CatBoost serving as the meta-learner.
- We use feature selection based on ANOVA F-scores and data imbalance handling with SMOTE, which significantly improves model generalization and classification performance.
- The model is highly accurate in predicting (86%), outperforming individual classifiers and similar state-of-the-art competitors on the PIMA Indian Diabetes dataset.
- In order to foster transparency and trustworthiness in clinical decision-making, we combine explainable AI methods (LIME and SHAP) to offer global and local interpretability of the model’s predictions.

The rest of this paper is structured as follows: Section reviews related work on diabetes prediction and ensemble learning techniques. Section explains the proposed method, such as data preprocessing, feature selection, model structure, and explainability methods. In Section, we present and discuss the experimental results, including an ablation study and performance assessment of the model. Lastly, Section concludes the research and presents directions for future work.

## Related work

The machine learning application for diabetes prediction has gained significant importance in recent years [13]. Several studies have explored diverse algorithms and datasets for diabetes prediction. Our literature review is divided into two parts: machine-learning-based approaches and deep-learning-based approaches.

### Machine learning approaches

Machine learning has been widely used for diabetes prediction due to its interpretability, transparency, and efficiency. Multiple algorithms have been explored for this task, such as Decision Tree, Naïve Bayes, Random Forest, Support Vector Machine, logistic regression, and have given promising results.

A study proposed an ensemble machine-learning framework for diabetes prediction [14]. They introduced a labeled diabetes prediction dataset (DDC) to address key challenges in diabetes prediction, including limited labeled data, missing values, and outliers. Different machine learning models, such as Decision Tree, Random Forest, XGBoost, and lightGBM, can be optimized through grid search. The model achieved better results with only four to five interpretable features (BMI, age, systolic and diastolic blood pressure, and occupation). This shows the potential of ensemble learning combined with effective preprocessing to enhance early diabetes detection.

Similarly, an ensemble-based framework for diabetes prediction was proposed [15]. They focused on methods that standardize, feature-select (via PCA, ICA, and correlation methods), and employ layered K-cross validation to improve model performance. Various classifiers, such as KNN, Decision Tree, Random Forest, AdaBoost, Naive Bayes, XGBoost, and Multilayer Perceptron, were evaluated individually and in combination. The weighted soft-voting ensemble approach, derived from the AUC of individual models, using AdaBoost and XGBoost, achieved excellent AUC and outperformed some cutting-edge methods.

A study [16] developed AdaptDiab which used adaptive combiner function to select the most informative features based on ensemble model characteristics. Furthermore, a study proposed a diabetic prediction model leveraging Machine Learning to improve the accuracy [17]. The framework evaluated the dataset’s performance across 4 classifiers: Random Forest, Support Vector Machine, Logistic Regression, and Naïve Bayes. effectively Other researchers focused on comparative analysis. A study [18] compared multiple algorithms on the PIMA dataset. The tree-based models achieved the highest classification accuracy.

Khaleel et al. [19] applied a variety of ML classifiers, including logistic regression, Naive Bayes, and K-Nearest Neighbor, on the Pima Indian Diabetes Data Set. LR outperformed other models, achieving 94% accuracy and demonstrating predictive efficiency for diabetes. Data standardization techniques such as Min-Max scaling were used to improve performance. A study integrated the models into a smart web application cite ahmed2021machine. Table 1 shows the performance of existing methods on diabetes prediction datasets.

**Table 1:** Performance of various machine learning models on diabetes prediction datasets. This table shows accuracy and whether explainable AI techniques (XAI) were used for each referenced model.

Ref	Year	Model	Dataset	Accuracy (%)	XAI
[14]	2022	Ensemble Learning (DT, RF, XGB, LGB)	DDC	73.50	No
[16]	2025	GNB, LR, RF, GB, MLP	PIMA	81	No
[18]	2023	NB, RF, J48	PIMA	79.57	No
[19]	2023	LR, NB, KNN	PIMA	80	No
[20]	2021	DT, NB, KNN, RF, GB, LR, SVM	PIMA Diabetes dataset 2	80.26 96.81	No
[21]	2024	LR, RF	Diabetes Binary Health Indicators BRFSS2015	84	Yes
[22]	2023	SVM, XGBoost, LR, RF, DT	PIMA, RTML	81	Yes

DDC = Diabetic Dataset Custom; PIMA = Pima Indians Diabetes dataset; XAI = Explainable Artificial Intelligence; DT = Decision Tree; RF = Random Forest; GB = Gradient Boosting; LR = Logistic Regression; NB = Naive Bayes; KNN = k-Nearest Neighbors; SVM = Support Vector Machine.

### Deep learning based approaches

Deep learning models have gained popularity due to their performance and their ability to capture non-linear patterns in medical data [23]. An improved Artificial neural network was proposed to enhance the accuracy of diabetes prediction [24]. Different data-preprocessing techniques, such as normalization and feature selection, were used. The model achieved superior learning efficiency and predictive accuracy compared to baselines by optimizing the neural network architecture and tuning hyperparameters effectively.

A study emphasized the growing global threat posed by diabetes and the urgent need for early detection [25]. They used the Indian diabetes dataset PIMA to evaluate the performance of their method. Another study introduced a machine learning pipeline and incorporated deep learning techniques to handle medical data effectively [26]. The XAI methods are categorized into post-hoc explanations and intrinsic approaches. The post-hoc explanations included SHAP and LIME, which explain the model after the fact. The intrinsic approaches focused on building interpretable models from the outset [27].

Researchers have turned their attention to model transparency. The studies [21,22] incorporated explainable AI methods such as LIME and SHAP. Another study has implemented ML algorithms, highlighting the importance of interpretability in models [28]. An early method [13] also validated the effectiveness of traditional models for diabetes prediction using clinical features. Furthermore, an explainable NLP model using Distill Bert and SHAP was developed to combat COVID-19 misinformation [29].

Similarly, a study [30] applied SHAP explanations to identify suicidal ideation during COVID-19.

Deep learning methods are growing for the diabetes prediction task [31]. Despite its significant adoption, a gap remains in its transparency and interpretability. In comparison, traditional machine learning models like decision trees and logistic regression are more transparent and easier to explain. But deep learning is evolving and dominating predictive modeling in health care; it becomes increasingly important to integrate explainability techniques such as LIME, SHAP, or attention mechanisms to enhance trust, accountability, and clinical acceptance.

## Proposed methodology

This study proposes a hybrid ensemble learning approach to address the challenges of imbalanced binary classification, as shown in Fig 5. Our model integrates data preprocessing, feature selection, SMOTE-based oversampling, and a stacking ensemble comprising multiple base learners and a powerful meta-learner. The Pima Indians Diabetes dataset is used as the benchmark to validate the proposed methodology.

### Dataset description

**Fig 2:**
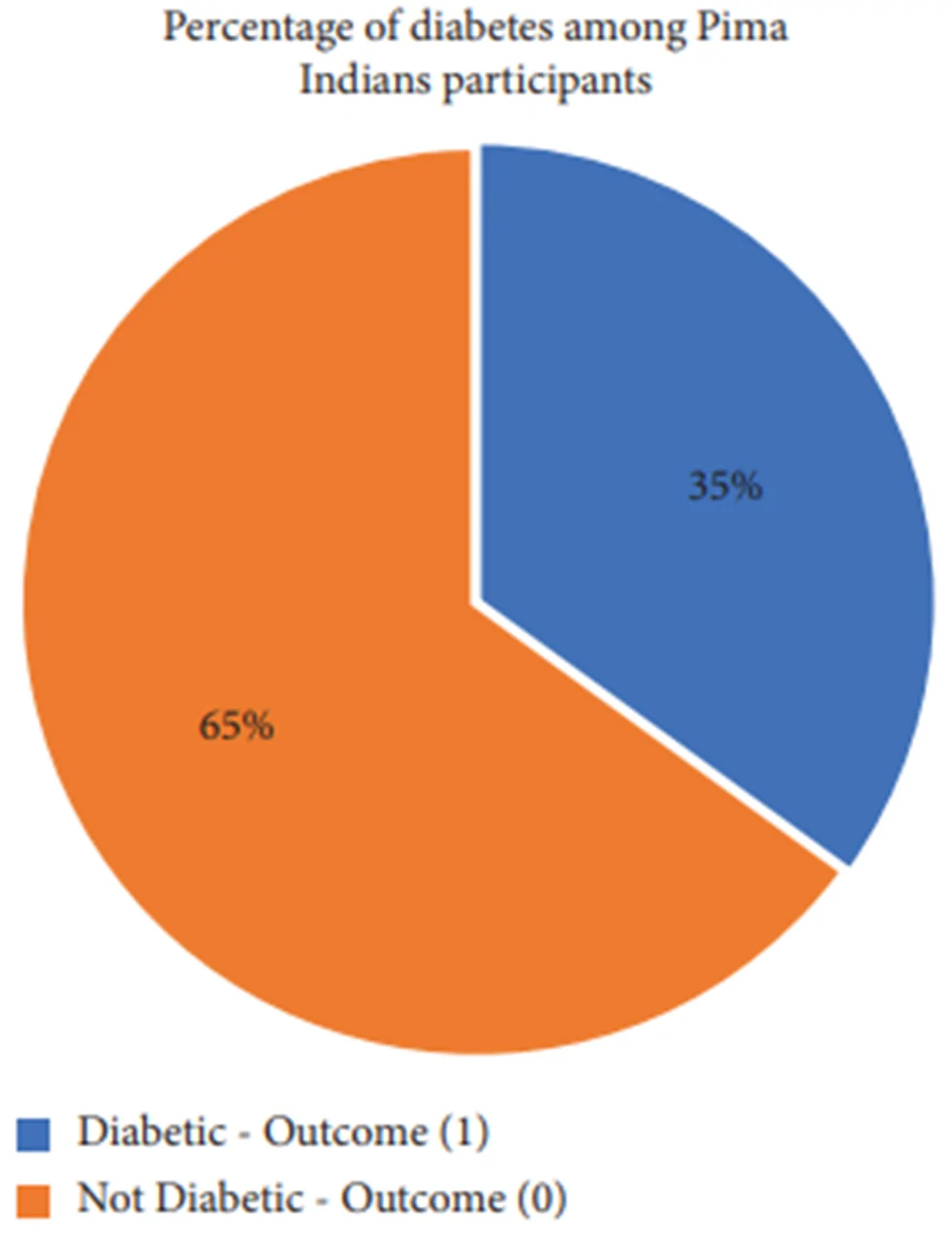
Distribution of diabetes outcomes among PIMA Indian Diabetes participants. The chart shows the ratio of diabetes vs. non-diabetic cases, demonstrating the natural class imbalance inherent in the dataset.

We used the PIMA Indians Diabetes dataset, a standard benchmark dataset for evaluating diabetes prediction models. It was obtained from Kaggle and contains 768 samples. The dataset includes eight clinical attributes: number of pregnancies, plasma glucose concentration, diastolic blood pressure, triceps skinfold thickness, two-hour serum insulin, body mass index (BMI), diabetes pedigree function, and age. The target variable, labeled as outcome, indicates the presence (1) or absence (0) of diabetes. The dataset is imbalanced as it contains 35% diabetic and 65% non-diabetic cases as can be seen in the Fig 2

**Fig 3:**
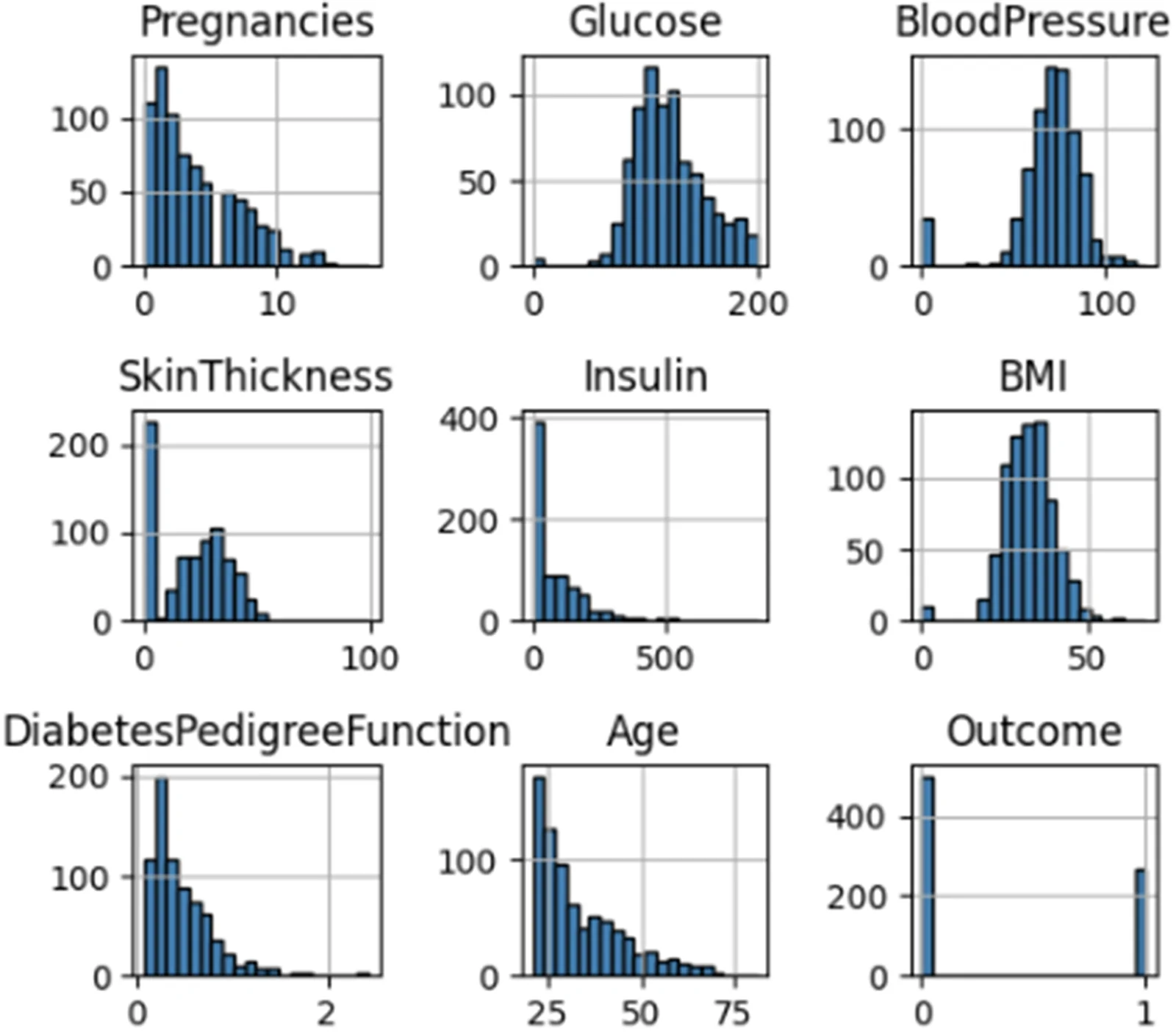
Histogram of the PIMA Indian Diabetes features. The plots show the distribution of important clinical features (e.g., glucose level, BMI, age), allowing the examination of data spread, skewness, and variability among participants.

Fig 3 presents the histogram of the dataset. It provides insights into the distribution of individual features. The visual pattern indicates that the dataset contains few outliers, indicating overall consistency.

**Fig 4:**
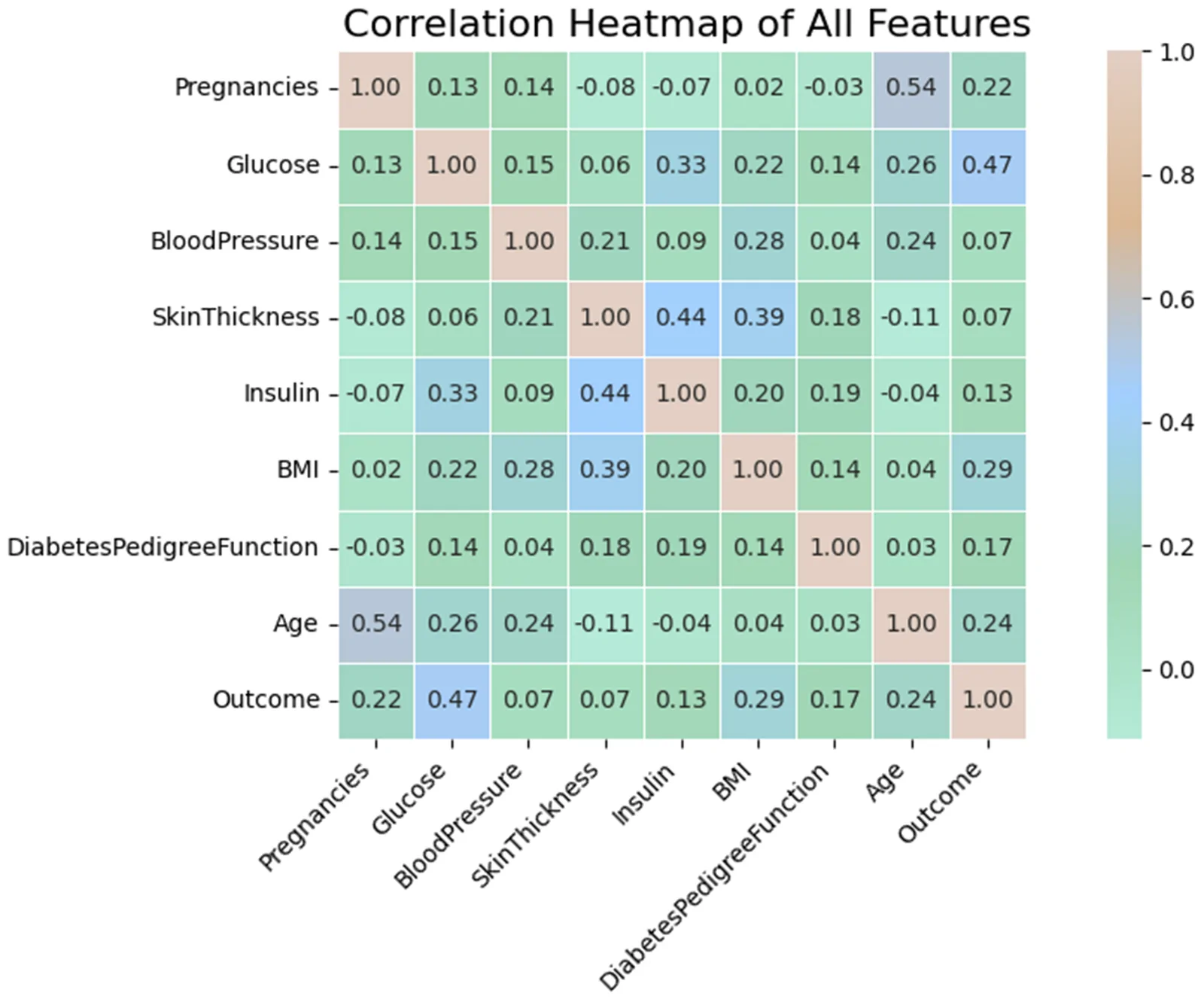
Correlation heatmap of all the features in the PIMA Indian Diabetes dataset. The blue–green–brown color intensity depicts the pairwise correlation strength and direction (from −1 to +1). Denser colors represent stronger correlations, with positive values appearing in brown and negative values in blue. Glucose and BMI exhibit stronger correlations with the diabetes outcome variable.

The correlation heatmap in Fig 4 provides a visual representation of the relationships between the dataset’s variables. No strong correlations are observed among the features. It highlights that the variables are largely independent.

Outlines the relationship network of all highlights in the Indian Diabetes dataset, using a color scale from cool blues to warm browns to represent the quality and direction of connections. Among all factors, glucose exhibits the strongest positive association with the target outcome (relationship coefficient: 0.47), indicating its strong significance for diabetes prediction. Other highlights, including BMI (0.29), pregnancies (0.22), and age (0.24), also show direct positive affiliations with the result.

The lattice reveals that numerous features exhibit weak intercorrelations, reducing concerns about multicollinearity and improving model stability. The clear, stylishly satisfying color palette enhances the interpretability of the lattice, supports the recognition of significant designs, and guides the determination of highlights, particularly by emphasizing glucose-related factors for future modeling endeavors.

### Data preprocessing

The dataset was sourced from Kaggle and includes eight input variables along with a binary target variable indicating the presence (1) or absence (0) of diabetes. Attributes such as glucose, blood pressure, skin thickness, insulin, and BMI had biologically implausible 0 values, which were handled as missing entries. To solve this problem, all zeros were replaced with NaN, and missing values were imputed with the median of each feature to preserve the underlying data distribution without bias.

Z-score standardization was used to center the features at zero and scale them to unit variance, making them comparable across scales. The dataset was split into training and test sets with a stratified 90–10 proportion to ensure an unbiased evaluation. The stratified method maintained the proportions of outcome classes in both sets, and the 90–10 split was chosen because the relatively small data set size (768 samples) enabled the model to learn strong patterns while retaining a representative test set for assessment.

Table 2 shows the results for the determined clinical skills before and after treatment. Before being used, these factors showed limited size and distribution, with large differences between imply and popular variance values across developments, as well as glucose, insulin, BMI and blood type. After preprocessing, Z-rating standardization converted all abilties to daily tests with suggested values based on zero and general deviations next to one. This normalization reduced the variability between skills, improved convergence, and ensured robust and unbiased model learning. This result indicates that the preprocessing pipeline standardized the data while maintaining the variability of clinical characteristics.

**Table 2:** Feature distribution before and after preprocessing.

Feature	Before Preprocessing (Mean ± Std)	After Preprocessing (Mean ± Std)
Glucose	120.89 ± 31.97	0.00 ± 1.00
BloodPressure	69.11 ± 19.36	0.00 ± 1.00
SkinThickness	20.54 ± 15.95	0.00 ± 1.00
Insulin	79.80 ± 115.24	0.00 ± 1.00
BMI	31.99 ± 7.88	0.00 ± 1.00
Age	33.24 ± 11.76	0.00 ± 1.00

### Feature selection

This study has enhanced the feature space to improve the model efficiency. We used the SelectKBest method with the ANOVA F-score to rank the features by importance. The top four features with the highest scores were selected for subsequent modeling.

### Handling class imbalance

There was a clear disparity in the dataset between the diabetes and non-diabetic classes. SMOTE generates synthetic samples of the minority class to create a more balanced training distribution, which helps reduce model bias and improve generalization.

We have proposed a stacking-based ensemble model. It improves overall performance by combining the strengths of several different classifiers. Stacking is a powerful ensemble learning technique that combines multiple base learners (level-0 models) and passes their outputs into a higher-level model (meta learner or level-1 model). The meta learner makes the final prediction.

### Base learner

This study includes three machine learning algorithms as base learners: Random Forest (RF), Gradient Boosting (GB), and Support Vector Classifier (SVC). Each of these classifiers uses a different learning strategy; they can identify distinct patterns and relationships in the data. Random Forest was used with 150 trees and a maximum depth of 8. Gradient Boosting is used to sequentially improve weak learners. It was employed with 150. Additionally, the Support Vector Classifier, a margin-based learner effective in high-dimensional spaces, was included with probability estimates enabled.

### Meta learner

The meta learner aggregates the base models’ predictions and produces the final classification. We have used CatBoostClassifier as a meta learner.CatBoost is a gradient boosting algorithm developed to handle categorical features natively and combat overfitting through built-in regularization techniques such as ordered boosting. Although our dataset is fully numeric, CatBoost’s robust implementation and strong handling of non-linearities make it a suitable choice as the final estimator.

### Model explainability

The interpretability of the ensemble model is enhanced by integrating two XAI techniques: LIME and SHAP. The ensemble model, especially with a meta learner such as CatBoost, offers strong predictive performance, but it inherently behaves as a black-box model. Therefore, to determine how individual features influence predictions and to gain insight into model behavior, post-hoc interpretability methods are used.

Local explanations are generated using LIME for individual predictions. LIME gives an explanation by estimating a complex model with an explanatory linear surrogate in the vicinity of each data example. This technique enabled us to imagine how each feature contributed positively or negatively to specific patients, making the model output more understandable for healthcare professionals.

On the other hand, Shap provides a global perspective by computing the average effect of each feature across all predictions using Shapley values from cooperative game theory. Shap not only provides continuity and local accuracy but also produces summary plots that help understand the inherent feature interactions.

## Results

This section presents the experimental evaluation of the proposed framework. It begins with the experimental setup and implementation details, followed by ANOVA F-test-based feature selection and correlation analysis of the selected features. Next, the performance of the proposed model is compared with baseline approaches. To enhance interpretability, SHAP and LIME are employed for global and local explanations, respectively. Finally, statistical analysis and ablation studies are conducted to validate the robustness and effectiveness of the proposed framework.

### Experimental setup

The proposed stack ensemble method achieves high accuracy and balanced classification performance. The stacking classifier consistently exceeded all baselines, including powerful individual learners such as Random Forest (RF), Gradient Boosting (GB), and Support Vector Classifier (SVC). A combination of several basic classifiers enables the model to generalize better, reducing compromises in distortion and variance.

Hyperparameter optimization is performed across all base models to improve generalization. Hyperparameter tuning is done to maximize the predictive accuracy of every base learner. For Random Forest and Gradient Boosting, the number of estimators, maximum depth, and learning rate are iteratively tuned manually based on validation accuracy. Likewise, the regularization parameter (C) of the Support Vector Classifier is empirically set to achieve bias–variance trade-off. For the CatBoost meta-learner, the depth, number of iterations, and learning rate are chosen via small-scale tests that optimize validation performance without overfitting. This is done to ensure computational tractability for datasets of this size, while still identifying successful setups for each model.

### Implementation details

The experiment is conducted by Google Colab in a Python-based environment. The implementation used Python 3.12.13 and the essential libraries such as NumPy 2.0.2, Pandas 2.2.2, Scikit-learn 1.6.1, and TensorFlow 2.19.0. The experiment is conducted by setting the random seed to ensure the reproducibility of the experiment. The necessary preprocessing techniques such as normalization and feature scaling have been applied. Table 3 shows the statement-tuned hyperparameters used for all algorithms in the stacking architecture.

**Table 3:** Hyperparameter settings for base and meta learners. This table lists all hyperparameters used for each model in the stacking ensemble.

Model	Hyperparameters
Random Forest	n_estimators=150, max_depth=8, random_state=42
Gradient Boosting	n_estimators=150, learning_rate=0.01, random_state=42
Support Vector Classifier	C = 1.2, gamma = scale, probability = True
CatBoost (Meta Learner)	learning_rate=0.0001, max_depth=5, iterations=200, random_state=42, verbose=0

This model is trained using a 90−10 split for training and testing. The test set was kept separate and wasn’t seen at all until the final evaluation. The stacking method used a split between training and testing, where the base learners’ predictions on the training data were used to train the meta-learner. Although using off-fold stacking might help reduce bias, the current setup was chosen to keep things simple for easier exploration. Smote is applied to balance the training data. After training, the model achieved excellent classification performance. The proposed ensemble framework is compared with individual base learners, including Random Forest, Gradient Boosting, and Support Vector Classifier. All models were trained on the Pima Indian Diabetes dataset after following the preprocessing steps: standard scaling, feature selection using ANOVA F-scores, and oversampling with SMOTE. ANOVA F test was applied using SelectKBest to evaluate the relationship between each feature and the target variable. The calculated F scores for all characteristics are presented in Table 4 Based on these results, the top four features (k = 4) were selected as they provided the greatest discriminative power and improved model performance during validation. Fig 6 illustrates the relationships among the selected features, pregnancies, glucose, BMI, and age used in the model. These pairwise correlations, ranging from −1 to +1, indicate varying degrees of positive association among the features. The strongest correlation is observed between pregnancies and age (0.54), suggesting that older people tend to report more pregnancies. Glucose shows moderate correlations with age (0.26), BMI (0.22), and pregnancies (0.13). Notably, BMI shows negligible correlations with pregnancies (0.02) and age (0.04), indicating that a few features contribute independent information.

**Table 4:** Anova f-scores of all features. This table lists f-scores of all features in the PIMA dataset.

Feature	F-Score
Glucose	213.16
BMI	108.03
Age	46.14
Pregnancies	39.12
DiabetesPedigreeFunction	34.71
Insulin	13.28
SkinThickness	4.30
BloodPressure	3.26

**Fig 5:**
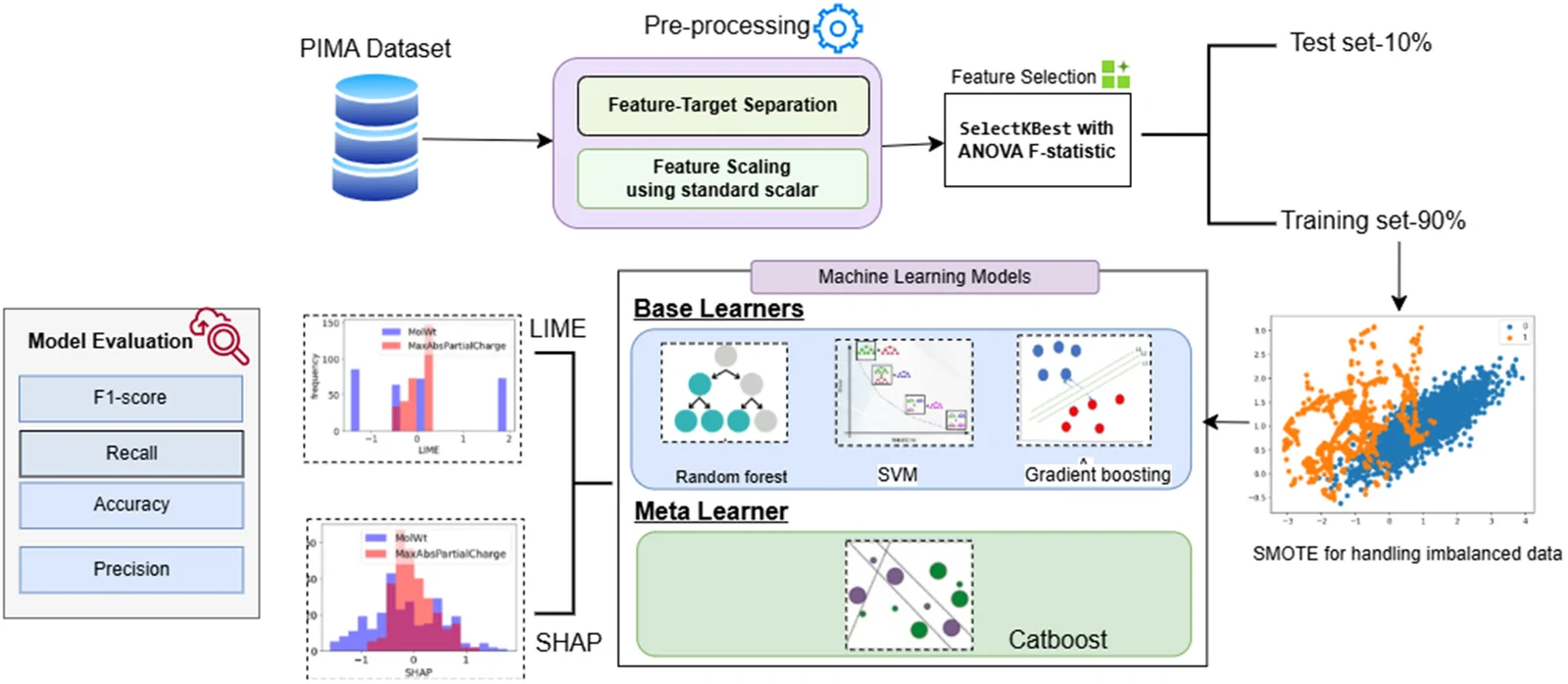
Hybrid stacking architecture for diabetes prediction. The proposed architecture combines several base learners (Support Vector Classifier, Random Forest, and Gradient Boosting) with CatBoost as the meta-learner. Data pre-processing involves scaling, feature selection, and SMOTE-based class balancing.

**Fig 6:**
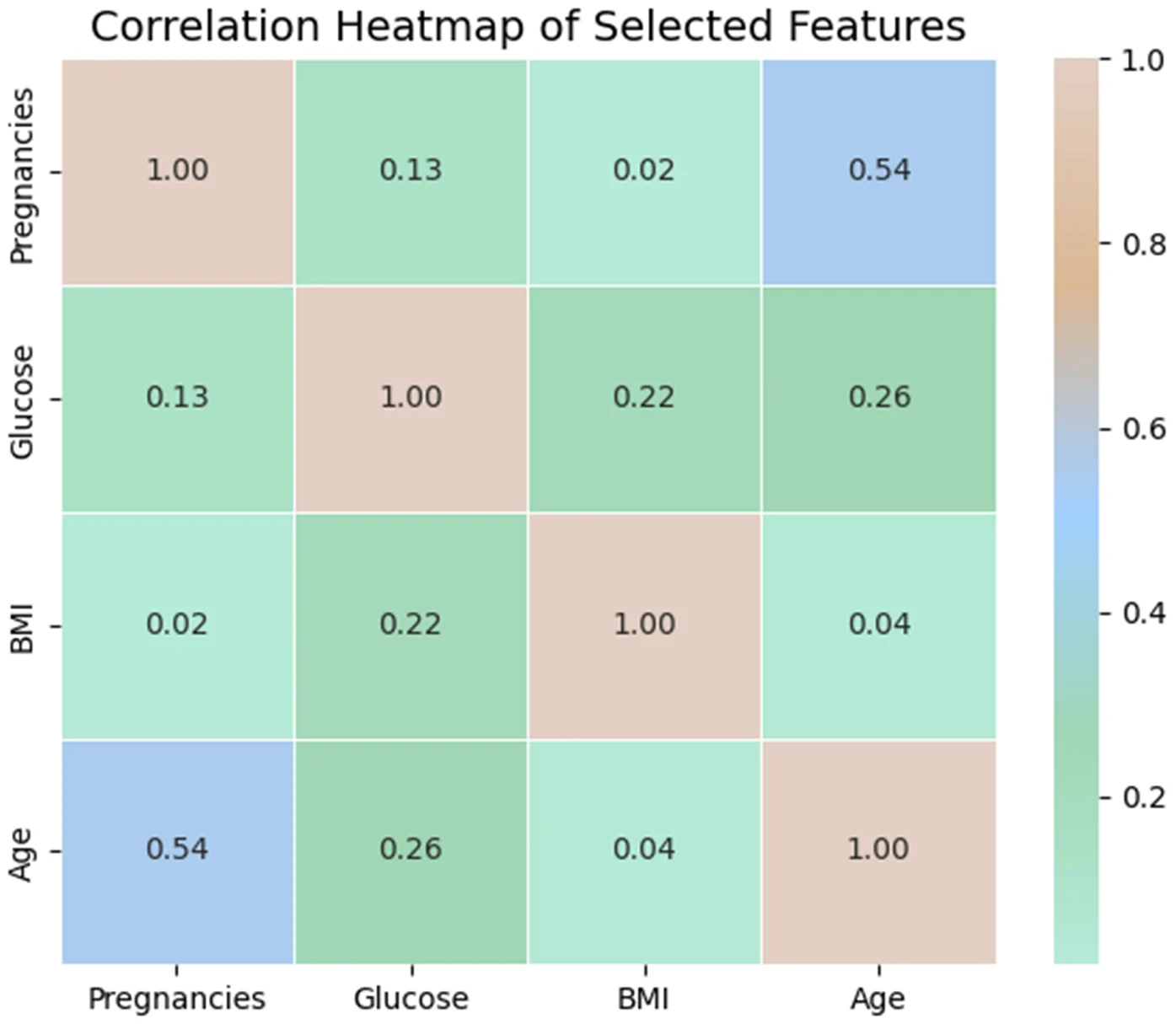
Heatmap of the correlation among the four chosen features (Pregnancies, Glucose, BMI, and Age) employed in the suggested model.

**Fig 7:**
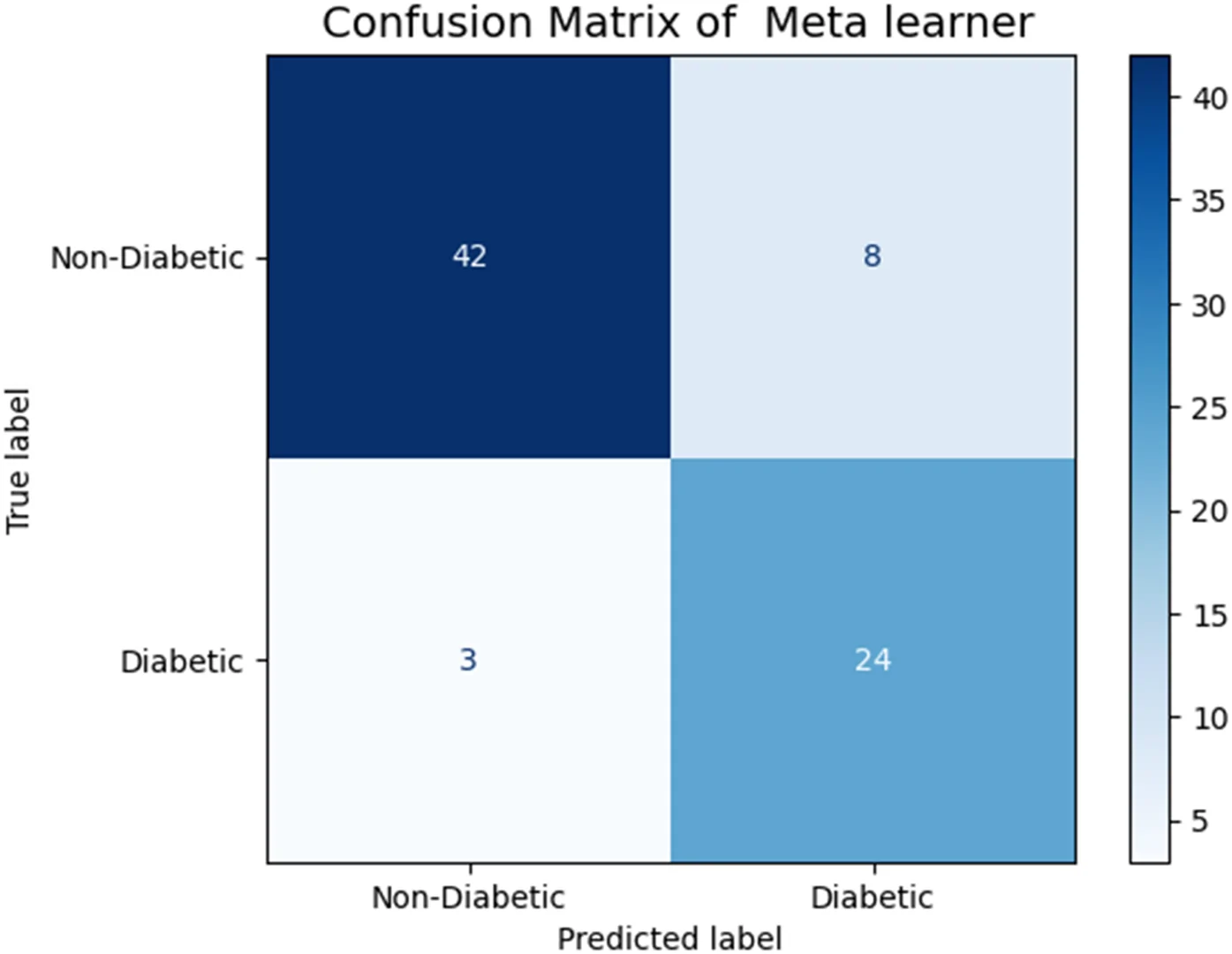
Confusion Matrix of Meta Learner.

Fig 7 shows the confusion matrix of the proposed stacked model that integrates predictions from random forests, gradient boosts, and SVM classifiers. The matrix shows the model’s effectiveness in classifying diabetic cases. The false class was relatively low, with 8 false positives and 3 false negatives. This performance reflects a balanced model with strong sensitivity and specificity, enabling accurate identification of diabetic patients while minimizing misclassifications among non-diabetic individuals.

The Fig 7 presents the confusion matrix of the meta learner. The model correctly classified 42 non-diabetic and 24 diabetic cases, while it misclassified 8 non-diabetic and 3 diabetic instances. These results indicate a strong balance between sensitivity and specificity, demonstrating the model’s robustness in handling both classes effectively. The relatively low number of false positives and false negatives supports the model’s practical utility for clinical decision-making.

**Fig 8:**
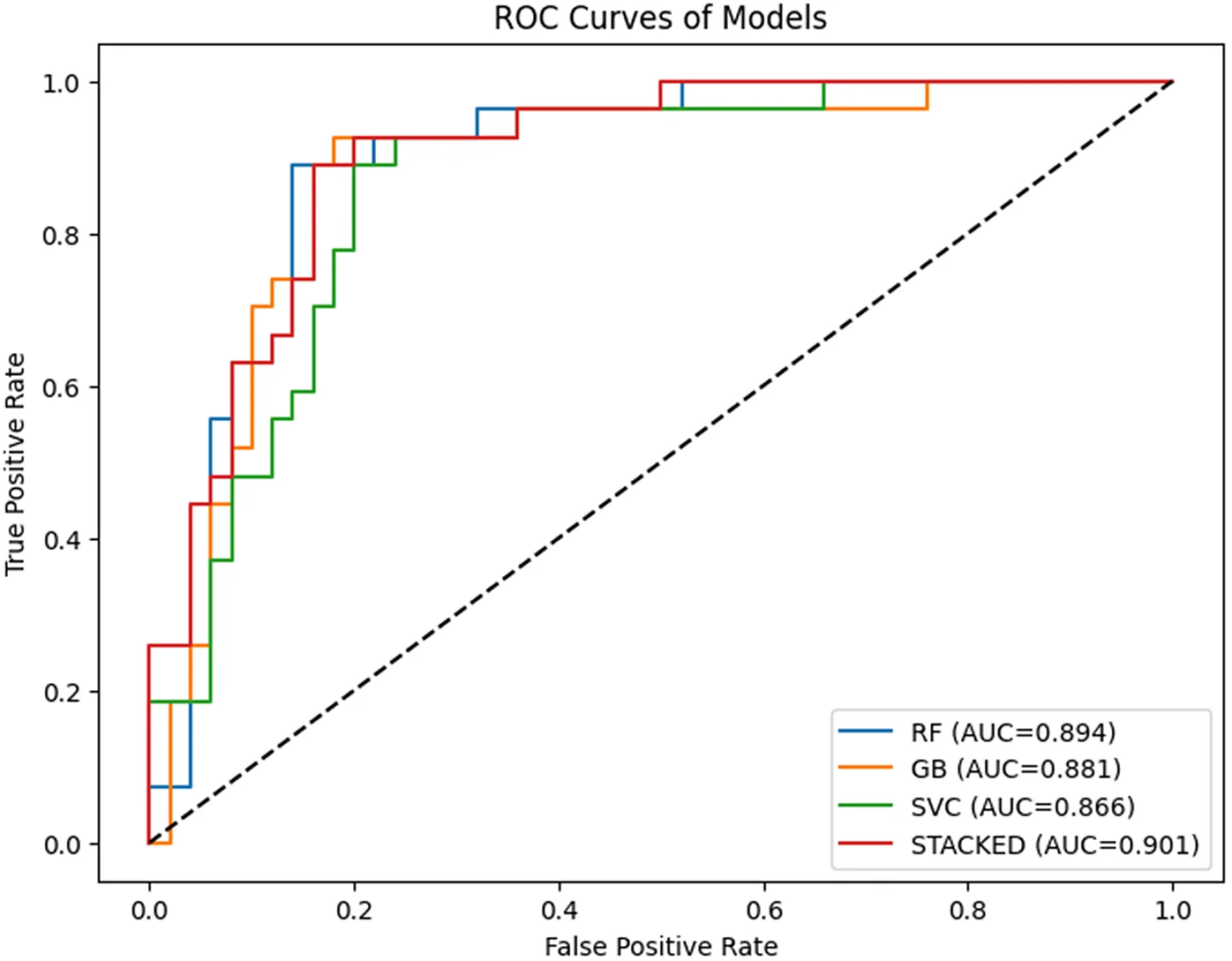
ROC and AUC of statistical significance testing between models.

The Receiver Operating Characteristic (ROC) curves demonstrate in Fig 8 a comparison of the classification performance of Random Forest, Gradient Boosting, Support Vector Classifier, and the proposed stacked model. The diagonal dotted line represents the performance of a random classifier, like AUC = 0.5, serving as a baseline. Curves closer to this line indicate weaker discriminative power, whereas those farther from it and closer to the top-left corner represent stronger predictive ability, with higher sensitivity and lower false-positive rates.

Although the stacked model’s curve briefly touches the diagonal at one threshold, its overall trajectory consistently lies above those of the other models, yielding the highest AUC value. In comparison with RF, GB, and SVC, the AUCs were 0.894, 0.881, and 0.866, respectively. This presents a despite minor fluctuations, the stacked model outperforms individual classifiers by effectively combining their strengths, thereby providing the most reliable classification performance.

**Fig 9:**
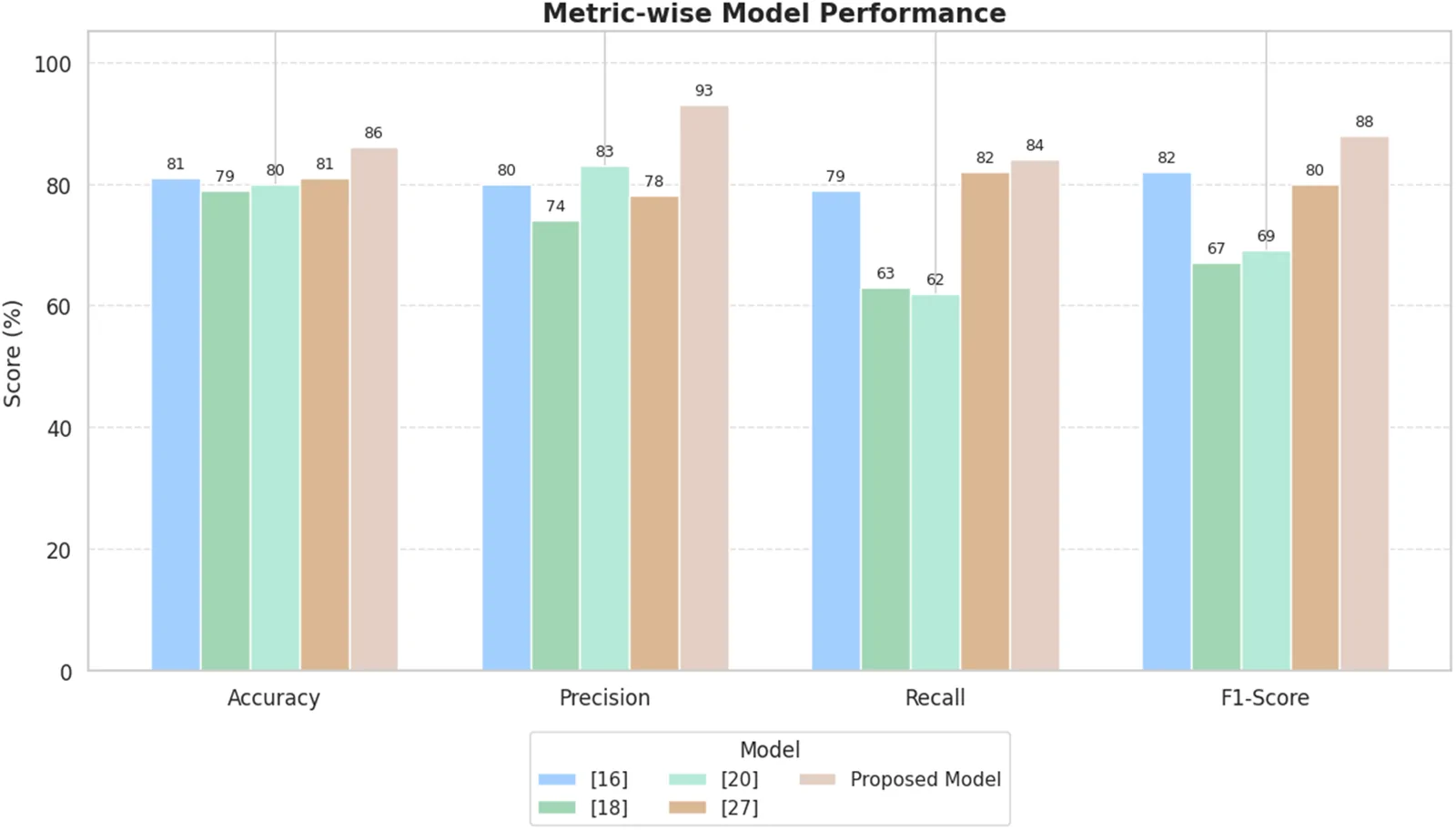
Comparison of classification performance according to Accuracy, Precision, Recall, and F1-Score. The results emphasize that the proposed hybrid stacking model outperforms the baseline models.

The proposed ensemble model exceeds the baseline methods across all metrics and achieved the highest precision (93%), showing reliable identification and best recall (84%) of diabetic cases in Fig 9.

Furthermore, the total accuracy was 86% and an F1 score of 88% was achieved, as can be seen in Table 5. This shows a robust balance between accuracy and recall. In contrast, the base model provided low recall and F1 scores, highlighting limited sensitivity and generalization.

**Table 5:** Performance comparison with existing state-of-the-art methods. This table compares the precision, recall, accuracy, and F1 score of the proposed model with previous approaches on the diabetes dataset.

Ref	Methodology	Precision (%)	Recall (%)	Accuracy (%)	F1-Score (%)
[16]	GNB, LR, RF, GB, MLP	80	79	81	82
[18]	NB, RF, J48	74	63	79	67
[20]	DT, NB, KNN, RF, GB, LR, SVM	83	62	80	69
[22]	SVM, XGBoost, LR, RF, DT	78	82	81	80
[32]	LR, RF, KNN	81	56	81	66
**Proposed Model**	**SVM, RF, GB, CatBoost**	**93**	**84**	**86**	**88**

All models evaluated on the same dataset split. Metrics expressed as percentages. GNB = Gaussian Naive Bayes; LR = Logistic Regression; RF = Random Forest; GB = Gradient Boosting; SVM = Support Vector Machine.

**Fig 10:**
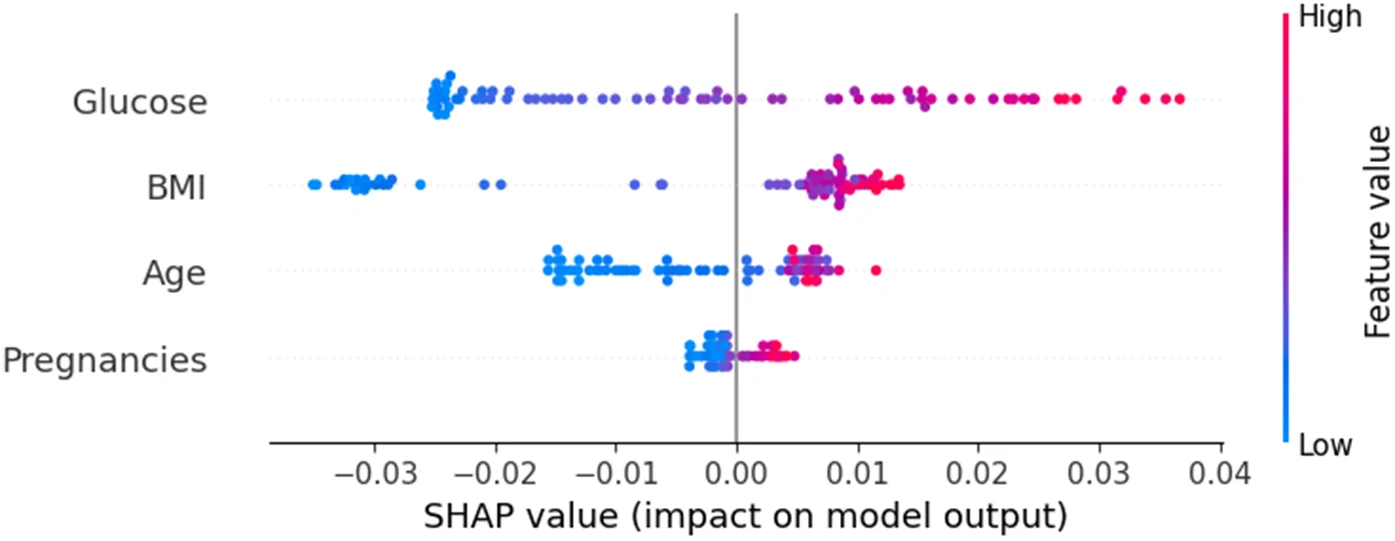
SHAP Summary Plot showing the global contribution of the top four features chosen (Glucose, BMI, Age, and Pregnancies) on model predictions, calculated across the entire PIMA Indian Diabetes dataset.

These results highlight the effectiveness of the proposed stack-based framework. In particular, it is a promising approach for clinical decision support in diabetes prediction, improving the accuracy of hunger class treatment and diagnosis. Fig 10 shows the SHAP summary plot. It shows the impact of the top 4 features, Glucose, BMI, Age, and Pregnancies, on the model’s output globally across the dataset.

Each point highlights the individual prediction, with the SHAP values on the x-axis showing the influence of that feature on the model’s final prediction. The color gradient shows the original feature values ranging from low (blue) to high (red). The figure shows that higher glucose levels increase the likelihood of a positive diabetes prediction. It is shown by the dense cluster of red points on the positive SHAP axis. BMI and age also influence the model’s prediction when their values are high, but the effect of pregnancies is mixed. It shows that both low and high values influence the prediction in opposite directions.

**Fig 11:**
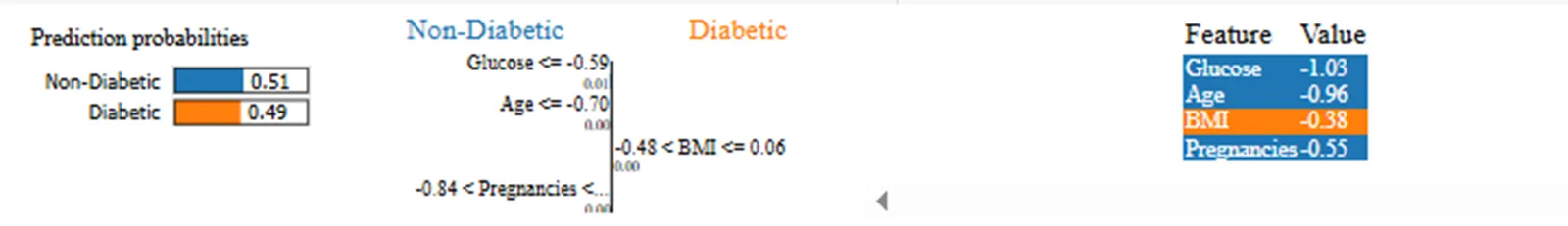
LIME local explanation for the True Positive prediction. The model accurately predicts that the instance is diabetic, with Glucose, Age, BMI, and Pregnancies as the most predictive features. The explanation outlines how the values of these features led to the positive prediction for this particular sample.

Fig 11 shows a lime explanation for a true positive prediction, where the model correctly identifies the case of diabetes. The prediction probabilities are almost balanced, with a slight bias towards the non-diabetic class (0.51 vs. 0.49). The explanation highlights that low values of glucose (−1.03) and age (−0.96) contributed most to the non-diabetic classification, whereas medium values of BMI (−0.38) and pregnancy (−0.55) slightly increased the likelihood of diabetes. This case indicates how the model integrates many weak signals to reach the correct final classification.

**Fig 12:**
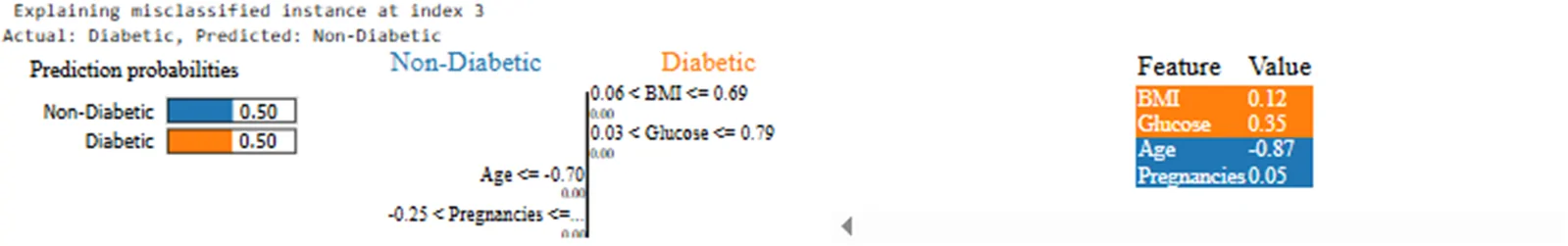
LIME local explanation of a False Positive prediction. The model mistakenly predicts a non-diabetic instance as diabetic. The local explanation indicates how Glucose, Age, BMI, and Pregnancies contributed to the decision and provides insights into why this misclassification occurred at the individual level.

Fig 12 presents a lime explanation for a false positive case, where the model misclassified a diabetic person as non-diabetic. The prediction probabilities are perfectly balanced (0.50 vs. 0.50), indicating high uncertainty. Lime mainly credits the decision for moderate values of glucose (0.35) and BMI (0.12), which push the prediction towards diabetes. However, low age (−0.87) and neutral pregnancy features(0.05) promote prediction towards the non-diabetic class, which eventually causes misclassifications. This figure highlights the model’s sensitivity to micro-feature combinations and areas where model calibration can be improved.

The proposed model was tested five times with random seeds to make sure it works well and gives consistent results as shown in Table 6. This way we can avoid getting results from just one test. The model that was stacked got an accuracy of 79.48% and the results were pretty consistent with a standard deviation of 5.16%. The accuracy varied a bit because the dataset was not very big and the data was split in ways. The model achieved an accuracy of 88.31%. This outcome is superior to the result achieved using a single test, which was 86% accuracy, 93% precision, 84% recall,and 88% F1-score. These results show that the proposed model is very good at making predictions. We saw some variation in the results, which shows that we need to check the statistics. Even with this variation the model performed consistently which is good. So the proposed model is not just accurate it is also reliable and works well which makes it good, for using in the world. Unlike studies that just do one test this study did many tests and checked the statistics, which makes the results more reliable and comprehensive.

**Table 6:** Statistical performance evaluation of proposed model.

Metric	Value
Number of Runs	5
Mean Accuracy	79.48%
Standard Deviation	5.16%
Maximum Accuracy	88.31%
Minimum Accuracy	74.03%
Best Single-Run Accuracy	86.00%
Precision (Best Run)	93%
Recall (Best Run)	84%
F1-Score (Best Run)	88%

### Ablation study

We conducted an ablation study to evaluate the contribution of individual base learners and the effect of the meta learner. The ablation study included various combinations of Random Forest (RF), Gradient Boosting (GB), and Support Vector Classifier (SVC). Both with and without CatBoost. Table 7 shows the results of experiments.

**Table 7:** Ablation study of accuracy with and without the meta learner (CatBoost). This table compares the performance of different base learner combinations, showing the impact of adding CatBoost as a meta learner on classification accuracy.

Exp.	Base Learners	CatBoost	Accuracy (%)
1	RF	No	78
2	RF	Yes	77
3	GB	No	77
4	GB	Yes	76
5	SVC	No	73
6	SVC	Yes	73
7	RF + GB	No	77
8	RF + GB	Yes	77
9	RF + SVC	No	73
10	RF + SVC	Yes	77
11	GB + SVC	No	75
12	GB + SVC	Yes	76
13	RF + GB + SVC	No	76
14	**RF + GB + SVC**	**Yes**	**86**

RF = Random Forest; GB = Gradient Boosting; SVC = Support Vector Classifier. The “CatBoost” column indicates whether the meta learner was included in each experiment. The bolded row highlights the best-performing configuration.

These experiments aim to analyze two key aspects: the effectiveness of each base learner when used independently or in combination, and the performance gain introduced by stacking the CatBoost meta learner.

### Individual learners

When Random Forest was tested alone, the accuracy was (78%), followed by Gradient Boosting (77%) and SVC (73%). Surprisingly, adding the meta-learner did not improve the performance of individual learners; rather, there was a reduction for RF (from 78% to 77%) and GB (from 77% to 76%), while that of SVC did not change. This shows that stacking itself is not always enough to enhance models with less diversity or lower individual performance.

### Pairwise combinations

In pairwise configurations (RF + GB, RF + SVC, GB + SVC), accuracies ranged between 73% and 77% without the meta learner. But when the meta learner is added, minor improvements are observed, most notably in the RF + SVC setup. The accuracy improved from 73% to 77%. It suggests that combining diverse models benefits more from stacking.

### Full ensemble

**Fig 13:**
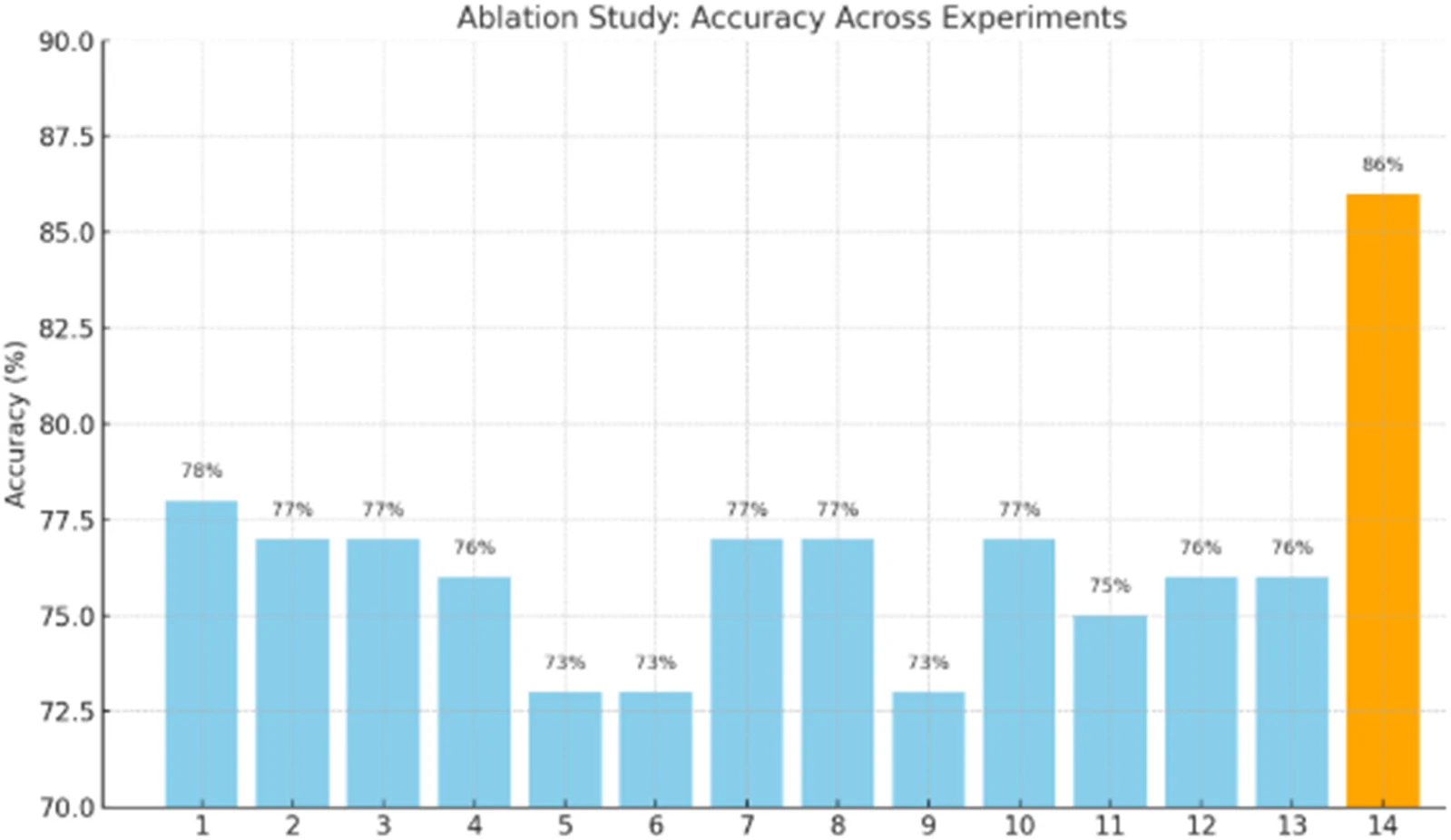
Ablation study: accuracy across different experimental settings. The highest accuracy of 86% is achieved in Experiment 14, whereas the other configurations range from 73% to 78%.

The significant improvement occurred when all three base models (RF, GB, and SVC) were used together with the meta-learner (CatBoost). On their own, each model had an accuracy of 76%, but when combined using CatBoost, the performance improved, reaching the highest accuracy of 86%, as shown in Fig 13. This happened because CatBoost learned from the predictions of all three models, identifying which model was best for each situation and correcting mistakes made by others. By leveraging the strengths of all three models, the stacked approach yielded better and more reliable predictions than any single model could achieve on its own.

## Conclusion

In this work, we developed a generalizable and robust ensemble machine learning framework for early diabetes detection using the PIMA Indian Diabetes dataset. The stacking ensemble model used Random Forest, Gradient Boosting, and Support Vector Classifier as base learners, CatBoost as the meta learner. Extensive preprocessing steps, including standardization, ANOVA F-score-based feature selection, and SMOTE data balancing, were used to improve the model’s performance. In this paper, our proposed method achieved an accuracy of 86% on the PIMA Indian Diabetes dataset, outperforming the individual classifier and the baselines. Various explainable AI methods, such as SHAP and LIME, were implemented to improve model transparency. By identifying key characteristics such as age, BMI, and glucose as major determinants of prediction, these techniques were used to provide both local and global interpretability. This not only supports model accountability but also facilitates clinical trust and adoption.

While this study used a single 90/10 train-test split and did not perform statistical significance tests, the findings indicate the promise of the suggested method. The stacking framework, while not entirely cross-validated, offers encouraging insights.

The stacking ensemble model works on the Pima Indians Diabetes Dataset. It has some problems.The Pima Indians Diabetes Dataset is small. Only has information from one group of people. This might make it hard to use the model for groups or in different hospitals.We used SMOTE to help with this. We chose the features and checked the model many times. There is still a chance that the model might not work well with new data because we got different results each time we ran it.The stacking ensemble model has not been tested on datasets.

This makes it hard to say for sure that the model is strong. SMOTE helps with class imbalance by making data. This fake data might not be exactly like real data.This could affect how well the stacking ensemble model works in life. We will work on these issues in the future to make the stacking ensemble model more useful and reliable.The stacking ensemble model works on the Pima Indians Diabetes Dataset. We need to make it better.

As future work, we would conduct a wider study by considering larger and more diversified diabetes linked datasets, aiming at a better overall generalizability, and will enhance these analyses through repeated statistical cross-validation assessments. We will also investigate the use of large language models (LLMs) to incorporate unstructured clinical data, past patient history, and electronic health records, leading to more context-aware and an expansive diabetes risk prediction.
